# De Novo Glioblastoma in the Territory of a Prior Middle Cerebral Artery Infarct

**DOI:** 10.1155/2013/356526

**Published:** 2013-10-10

**Authors:** Teresa J. Wojtasiewicz, Andrew F. Ducruet, Sonal S. Noticewala, Peter Canoll, Guy M. McKhann

**Affiliations:** ^1^Department of Neurological Surgery, College of Physicians and Surgeons, Neurological Institute of New York, Columbia University, 710 W 168th Street, New York, NY 10032, USA; ^2^Department of Pathology, Columbia University, College of Physicians and Surgeons, 1130 St. Nicholas Avenue, New York, NY 10032, USA

## Abstract

We report a case of a patient who developed glioblastoma in the territory of a previous infarction. Two years after an ischemic stroke, the patient presented with a cystic, necrotic, and heterogeneously enhancing mass. Open biopsy and debulking of the mass with histological analysis revealed the mass to be glioblastoma. Though several cases of posttraumatic GBM have been reported, this is the first proposed case of GBM after an ischemic stroke. From this case, we suggest that the ischemic stroke, like other forms of cortical injury, may predispose to glioblastoma formation.

## 1. Introduction

Previous reports have suggested that glioblastoma (GBM) may arise from areas of gliosis resulting from traumatic brain injury, chronic abscess, or surgical resection [1–4]. The process of reactive gliosis that follows such injuries may increase the chance of malignant transformation. The mechanisms underlying this process remain unclear. Here, we present a patient who developed GBM two years after an ischemic infarction. We propose that this GBM developed in the region of previous infarction and review the existing evidence of posttraumatic tumorigenesis.

## 2. Case Report

### 2.1. Initial Presentation

**Figure 1:**
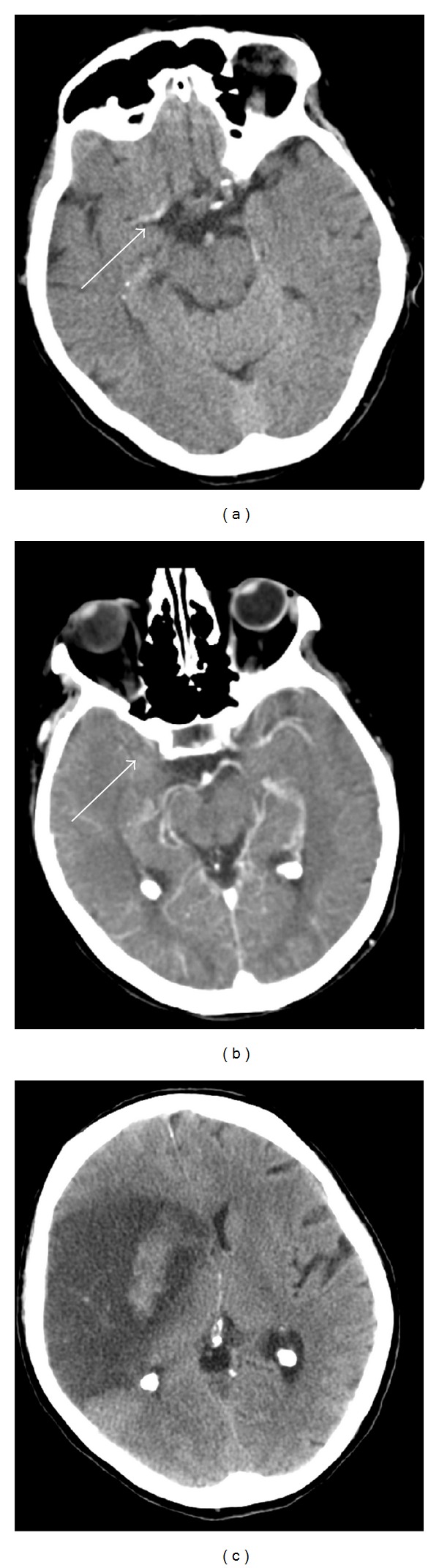
Imaging of initial ischemic stroke. (a) CT without contrast, showing dense right MCA (white solid arrow) and loss of differentiation of the grey-white junction on the right. (b) CT angiography, revealing occlusion of the right MCA (white solid arrow). (c) CT without contrast, showing subsequent hemorrhagic transformation of the infarct.

A 73-year-old woman with a history of mechanical aortic valve replacement and atrial fibrillation, maintained on warfarin, initially presented with acute left-sided hemiparesis and a right gaze deviation in the setting of a subtherapeutic prothrombin time/international normalized ratio (PT/INR). Head computed tomography (CT) revealed loss of differentiation of the grey-white junction in the right insula and lateral basal ganglia with densities in the right middle cerebral artery (MCA) and right internal carotid artery (ICA) (Figure 1(a)). Concurrent CT angiography (CTA) revealed an occlusion of the supraclinoid segment of the right ICA extending to the M1 segment of the MCA (Figure 1(b)). Despite intravenous tissue plasminogen activator (tPa), intra-arterial urokinase, and attempted mechanical thrombectomy, her occlusion remained. 

Serial head CT scans obtained over the next several days revealed an evolving right MCA territory infarction extending to the right temporal pole, frontal operculum, and basal ganglia, as well as a hemorrhagic conversion of this infarct (Figure 1(c)). She was ultimately discharged to rehabilitation on day 17 with a profound left hemiparesis.

### 2.2. Subsequent Imaging

**Figure 2:**
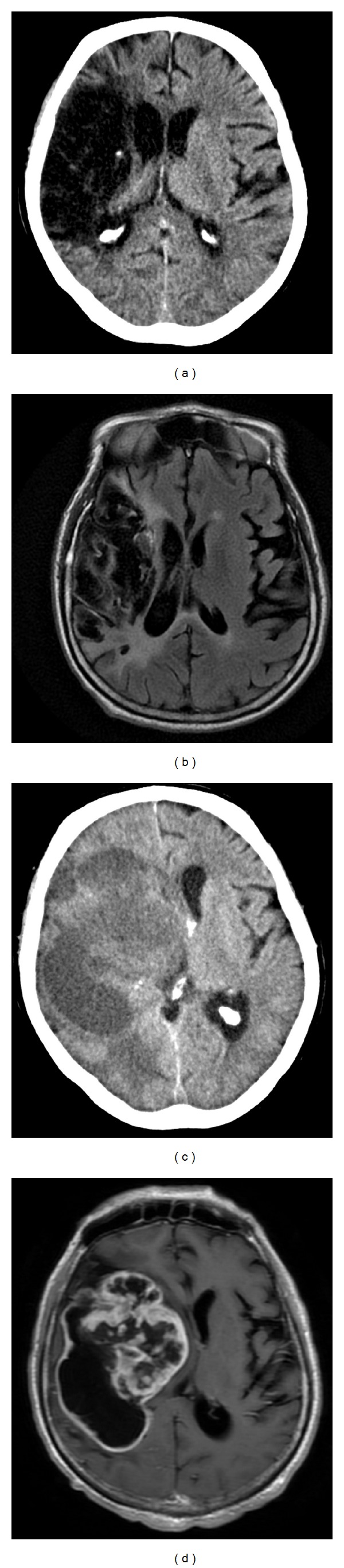
Evolution of gliotic scar after ischemic stroke. (a) CT without contrast 17 months after ischemic stroke, showing a gliotic scar. (b) MRI FLAIR sequence 17 months after ischemic stroke, showing gliotic scar within the previously infarcted region. (c) CT without contrast two years after ischemic stroke, showing a dense, cystic area in the right frontal, temporal, and parietal lobes, corresponding to the region of the prior infarct. (d) MRI T1 sequence after gadolinium contrast two years after ischemic stroke, showing contrast-enhancing, necrotic mass in the previously infarcted region.

Seventeen months after infarction, head CT revealed a well-defined area of encephalomalacia in the right MCA distribution accompanied by ex vacuo dilation of the left lateral and third ventricle (Figure 2(a)). MRI at that time similarly revealed encephalomalacia of the right MCA territory, with extensive leukomalacia in the area of previous infarction (Figure 2(b)).

### 2.3. Presentation with Cystic, Necrotic Mass

Two years following her initial ischemic stroke, the patient presented with a four-day history of severe headaches and increased somnolence. Head CT revealed a large, heterogeneously dense, and cystic area spanning the right frontal, temporal, and parietal lobes. This corresponded to the previously MCA infarcted territory (Figure 2(c)). MRI with gadolinium contrast showed a necrotic, heterogeneously enhancing mass within the prior infarct that was causing a significant right-to-left midline shift with effacement of the right lateral ventricle (Figure 2(d)). A decision was made in conjunction with the family's wishes to perform an open biopsy with subtotal decompression of the cystic component of this mass.

### 2.4. Biopsy and Subtotal Resection/Decompression

A right-sided frontotemporal craniotomy was performed using frameless stereotactic guidance, and the wall of the cystic component of the mass was biopsied. Intraoperative pathology was consistent with glioblastoma. Subtotal debulking proceeded, including drainage of the accessible cystic components of the tumor. Her postoperative course was uneventful. She was discharged home on postoperative day 13 and scheduled to follow up with a neuro-oncologist for consideration of adjuvant treatment options. Her family chose palliative care for her. She expired from medical complications approximately 5 weeks after the diagnosis of GBM.

### 2.5. Pathologic Analysis

**Figure 3:**
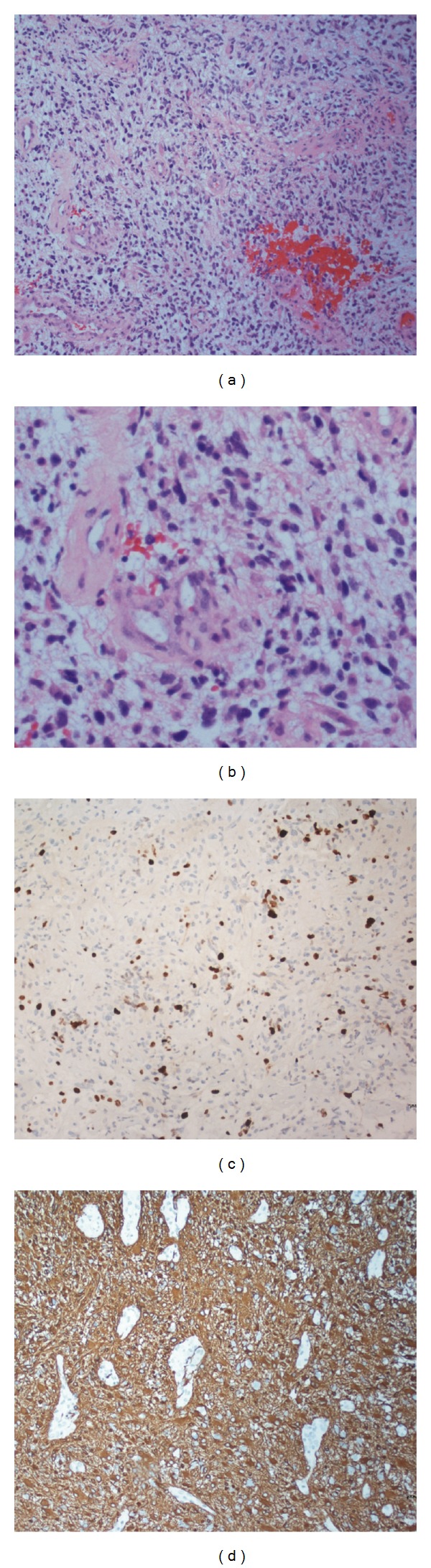
Microscopic image of the patient's GBM. (a) Low-power micrograph of hemotoxylin and eosin staining, showing cellular atypia, glomeruloid vascular pattern, and areas of tumor necrosis diagnostic of GBM. (b) High-power micrograph showing atypical, hyperchromatic nuclei and numerous mitotic figures. (c) Low-power micrograph showing numerous cells staining positive for Ki67, a marker of cell proliferation. (d) Low-power micrograph showing staining for glial fibrillary acidic protein (GFAP).

Histological analysis of the tumor showed pleomorphic neoplastic cells with irregular hyperchromatic nuclei, numerous mitotic figures, robust vascular proliferation, and areas of tumor necrosis (Figure 3). These features are diagnostic for GBM (WHO grade IV). Immunostaining was strongly positive for glial fibrillary acidic protein (GFAP), negative for isocitrate dehydrogenase 1 (IDH1), and negative for p53 in the majority of cells. Furthermore, immunostaining was weakly positive for phosphatase and tensin homolog (PTEN) in tumor cells and strongly positive in endothelial cells; weakly focally positive for epidermal growth factor receptor (EGFR); and positive for Ki67 in approximately 15–20% of cell nuclei.

## 3. Discussion

This is the first case to propose that GBM could develop within the territory of a previous ischemic infarct. The distribution of postinfarction encephalomalacia corresponds to the distribution of the subsequent GBM, suggesting tumor arising within infarcted tissue (Figure 2). The lack of evidence of GBM in prior imaging (Figure 1) suggests that the GBM developed after the infarction, potentially arising from the glial scar. There are rare reports of patients with an established diagnosis of ischemic stroke and no radiographic evidence of a tumor which developed GBM within 7–10 months of their infarction [5, 6]. One case describes a misdiagnosed “early stage” GBM in a patient with a history of ischemic stroke, which could have been a GBM that developed in an old infarct [6]. Another report describes a patient with no stroke risk factors who developed an MCA infarct, had no evidence of a mass on MRI three months after the stroke, and developed a contrast-enhancing mass seven months after the stroke [5]. In these previous reports, the authors speculate that subclinical GBM, which has been shown to be capable of invading arterial walls, was the etiology of the reported ischemic injury [5–7]. However, the correlation between the initial infarct and the subsequent GBM in these cases is not completely clear. These may be other cases of malignant transformation of cerebral infarcts. In the case we present here, the patient's presenting symptoms, radiological findings, and clinical progression are consistent with an ischemic stroke caused by cardiac thrombi, rather than subclinical GBM. Furthermore, if any GBM had been present at the time of initial ischemic injury, a latency of two years before development of symptomatic tumor would have been highly unlikely—the putative reports of subclinical GBM indicate a latency of less than a year before development of symptoms.

The mechanism of development of a postischemic GBM may be similar to the mechanism of posttraumatic GBM. Glial malignancies have been reported after various forms of brain injury, including penetrating head injury, cerebral contusion, and previous surgical resection [1–4]. Neural trauma following brain injury, including ischemic stroke, results in astrocyte activation and subsequent reactive gliosis [8, 9]. Gliosis may lead to tumorigenesis, as both reactive astrocytes and glial progenitor cells are proposed origins for GBM [8, 10, 11]. However, most of gliomas develop without an identifiable precipitating event, and the mechanisms of malignant transformation in glial neoplasms are not well understood. Epidemiological studies have not definitively shown an increased incidence of brain tumors in patients with head injuries [12, 13]. Epidemiological studies of patients who have had ischemic strokes also do not show an increased incidence of malignant glioma [4]. However, most ischemic white matter disease is clinically unrecognized, with 92% of elderly individuals (60–90 years old) showing some degree of white matter abnormality and 24% having at least one prior cerebral infarction [14, 15]. We speculate that a small ischemic lesion could lead to the development of GBM, just as a large MCA infarction appears to have led to GBM development in the case we presented here, but the role of the initial ischemic event in tumorigenesis would not be apparent. One possibility is that reactive gliosis caused by CNS injury, including ischemia, is involved in the pathological process of glioma formation.

 The pathology specimen obtained from our patient represents the end-stage of malignant transformation. Though tissue samples of intermediate stages of malignant transformation would be necessary to completely elucidate the mechanisms of GBM development, we have proposed hypothetical mechanisms based on experimental data. We theorize that there was proliferation of astrocytes and glial progenitor cells after this patient's ischemic stroke, based on strong evidence that ischemia potentiates gliosis and proliferation of glial progenitor cells [9, 16, 17]. Without tissue samples from earlier stages of the malignant transformation, the hypothesized mechanisms of tumorigenesis remain speculative.

## 4. Conclusions

This is the first reported, pathologically proven case of GBM developing in a territory of a previous cerebral infarction. Other reports have shown that many forms of brain injury promote glial scarring which may induce GBM formation [1–3, 18, 19]. From the case described here, we postulate that ischemic stroke, like other forms of cortical injury, may predispose to the formation of GBM [9].
